# Strangulated Hernia—Treatment by Hypodermic Injections of Morphia Hydrochloras

**Published:** 1878-09-20

**Authors:** G. H. Chapman

**Affiliations:** Ill.


### STRANGULATED HERNIA—TREATMENT BY HYPODERMIC INJECTIONS OF MORPHIA HYDROCHLORAS.

By G. H. Chapman, M.D., Ill.

In the month of October, 1876, while practicing in Hudson, Mich., with my father, Dr. Geo. Chapman, the following case presented itself, which, with others since treated in a similar manner, seems to me of sufficient interest to report. I therefore report them, hoping it may meet the approval of some of my professional friends, and possibly save some suffering mortal hours of agony, if not operation, in some instances:

*Case* I.—John R., a farmer, aged 61, of medium height, robust and muscular, came into the office, having an oblique inguinal hernia of the right side, which had "come down," and, contrary to its usual custom, completely resisted his personal efforts to reduce it. He had been troubled with it for several years, but had worn a truss. Upon examination we found the tumor of a hard, globular character, dark-red, almost purple color, and about the size of a common lemon. All our efforts to reduce it by taxis, with fomentations, tractile method (inverting the patient), &c., proved unavailing after two hours' hard work.

We debated the question of using chloroform, as our patient had an intermittent pulse; still, feared we might be obliged to resort to it, and possibly to an operation, as the patient now vomited at each effort at taxis.

At this form, my father called to mind the fact of having read, somewhere, of the hypodermic injection of a full dose of hydrochlorate of morphia in the reduction of a dislocated humerus, and said: "If useful in that case, why not in this?"

Acting on this suggestion, I immediately injected ⅓ grain of the drug in the outer side of the thigh, about six inches from the tumor, and waited patiently ten minutes, till the patient said, "I have no pain."

We now raised him so that the hips were about fifteen inches higher than his head and shoulders, and resumed taxis, when, to our surprise and infinite relief, at the first attempt the tumor returned to the abdomen with an audible click.

There was some tenderness and inflammation in the injured region for a few days, and, after this subsided, a suitable truss was applied, and no more trouble was experienced.

*Case* II.—About one month after the preceding case I was summoned to attend a young man five miles in the country—a farmer, aged twenty.

Upon examination, found an oblique inguinal hernia of the right side, extending into the scrotum, about the size of a Bartlett pear, of a purplish color, with intense pains at abdominal rings. This patient had often reduced the hernia himself, but in this instance had worked *twelve hours* without success, and was now vomiting at each effort at taxis. I immediately injected ⅓ grain of morphia hydrochloras into the thigh, and waited fifteen minutes, when I had the satisfaction of having the tumor return to the abdomen upon my first attempt at reduction by taxis, with the same audible click as in the former case.

*Case* III, occurred in Grand Crossing, Ill. Was called January 9, 1878, to see C. W., aged 29, and found an inguinal hernia similar to No. II, as to present condition, time of duration, patient, vomiting, &c.

I administered in the thigh, as before, ¼ grain morphia hydrochloras, and succeeded in reducing the tumor in half an hour.

I lay the lack of *immediate* success in this case to the smaller dose of morphia used.

This patient neglected to have a truss adjusted, consequently he again presented himself, in the same condition, on the 3d of March. I had no hydrochlorate at this time, and could get none in town; I therefore injected ⅓ grain of the sulphate, and attempted reduction by taxis and tractile method, without success. I then administered chloroform, which increased the vomiting, and made a bad matter worse. I now injected ¼ grain of morphia as a second injection, and applied fomentations for an hour; then resorted to taxis once more, and, after considerable effort, succeeded in relieving my patient.

Hydrochlorate of morphia, though used far less frequently in this country than the sulphate, is received with greater favor in Great Britain. My experience with the drug leads me to assert that it possesses peculiar advantages over the sulphate in many cases, especially where we seek to relieve spasm, as is shown in Case III. I have also observed its superiority in asthma. One case particularly I now recall, where the druggist substituted sulphate for muriate (as he afterwards admitted), without consent, and no relief was obtained. I had the prescription re-compounded properly, and secured relief on administering the first dose.

I further find it less liable to cause the peculiar nausea and "heaviness of the head" so often complained of after taking the sulphate.

Its advantages in cases like the above is self-evident, as in the after-treatment of such cases, as well as in dislocations, we almost invariably are obliged to administer an anodyne. By administering it in a maximum dose (which I consider essential for the best results) before operation, we may avoid the dangers of chloroform, securing equally good results. Will it stand the test?—*Mich. Med. News.*

